# Ambulatory daytime blood pressure versus tonometric blood pressure measurements in the laboratory: effect of posture

**DOI:** 10.1097/MBP.0000000000000651

**Published:** 2023-06-08

**Authors:** Emmi Värri, Lauri Suojanen, Jenni K. Koskela, Manoj K. Choudhary, Antti Tikkakoski, Mika Kähönen, Pasi I. Nevalainen, Jukka Mustonen, Ilkka Pörsti

**Affiliations:** a Faculty of Medicine and Health Technology, Tampere University, Departments of; b Internal Medicine; c Clinical Physiology, Tampere University Hospital, Tampere, Finland

**Keywords:** ambulatory recording, blood pressure, head-up tilt, hypertension, laboratory measurement

## Abstract

To compare blood pressure (BP) in tonometric radial artery recordings during passive head-up tilt with ambulatory recordings and evaluate possible laboratory cutoff values for hypertension.

**Methods:**

Laboratory BP and ambulatory BP were recorded in normotensive (n = 69), unmedicated hypertensive (n = 190), and medicated hypertensive (n = 151) subjects.

**Results:**

Mean age was 50.2 years, BMI 27.7 kg/m^2^, ambulatory daytime BP 139/87 mmHg, and 276 were male (65%). As supine-to-upright changes in SBP ranged from −52 to +30 mmHg, and in DBP from -21 to +32 mmHg, the mean values of BP supine and upright measurements were compared with ambulatory BP. The mean(supine+upright) systolic laboratory BP was corresponding to ambulatory level (difference +1 mmHg), while mean(supine+upright) DBP was 4 mmHg lower (*P* < 0.05) than ambulatory value. Correlograms indicated that laboratory 136/82 mmHg corresponded to ambulatory 135/85 mmHg. When compared with ambulatory 135/85 mmHg, the sensitivity and specificity of laboratory 136/82 mmHg to define hypertension were 71.5% and 77.3% for SBP, and 71.7% and 72.8%, for DBP, respectively. The laboratory cutoff 136/82 mmHg classified 311/410 subjects similarly to ambulatory BP as normotensive or hypertensive, 68 were hypertensive only in ambulatory, while 31 were hypertensive only in laboratory measurements.

**Conclusion:**

BP responses to upright posture were variable. When compared with ambulatory BP, mean(supine+upright) laboratory cutoff 136/82 mmHg classified 76% of subjects similarly as normotensive or hypertensive. In the remaining 24% the discordant results may be attributed to white-coat or masked hypertension, or higher physical activity during out-of-office recordings.

## Introduction

To diagnose hypertension in clinical practice, the gold standard is ambulatory blood pressure (BP) monitoring where the cutoff point for hypertension in daytime recordings is 135/85 mmHg, in nighttime recordings 120/70 mmHg, and in 24-hour recordings 130/80 mmHg [1]. As ambulatory BP monitoring requires resources and may cause discomfort, the diagnosis of hypertension is commonly achieved by repeated home BP measurements. The cutoff point of hypertension in home measurements is also 135/85 mmHg [1].

BP is characterized by high variability, and based on wrist-worn tracker analyses of heart rate [2], a single day contains about ~100 000 SBP and DBP values. The standard home recording of BP is carried out in the seated position [1], and excludes information about posture-related changes in BP. The cuff-based BP measurement was recently reported to underestimate SBP by 6 mmHg and overestimate DBP by 6 mmHg, and these effects were progressively increased with advancing age [3,4]. The authors suggested that more personalized methods of BP measurements should be developed [3]. The common phenotypes white-coat hypertension and masked hypertension add complexity to the diagnosis of hypertension and evaluation of cardiovascular risk [5,6]. Even moderate changes in BP in the absence of hypertension are associated with cardiovascular risk, and recent evidence suggests that the risk increases gradually from SBP level of 90 mmHg [7,8].

The study of hemodynamic changes associated with hypertension is usually carried out in the laboratory [9–12]. For instance, a wealth of information regarding wave reflections and arterial stiffness has been achieved under laboratory conditions [10,13–16]. The inclusion of head-up tilt in the measurements has provided information about the influence of posture on cardiovascular regulation [10,12,17,18]. The BP response to upright posture is variable, ranging from orthostatic hypotension to orthostatic hypertension, whilst the divergent BP reactions to upright posture have also an impact on patient prognosis [19–23]. The hemodynamic changes in response to upright posture are also influenced by antihypertensive medication [10], cardiovascular disease [24], age [25], and sex [18].

As a major proportion of research focusing on the hemodynamics of hypertension has been performed under laboratory conditions, our objective was to compare the results of tonometric laboratory BP recordings versus ambulatory daytime BP measurements and evaluate possible laboratory cutoff values for hypertension. This information mainly serves research purposes but may be helpful in the estimation of the reliability and validity of the laboratory measurements for real-life situations. We also compared supine and upright BP during laboratory measurements with ambulatory daytime BP and evaluated the accuracy of the laboratory measurements to classify patients as normotensive and hypertensive.

## Methods

### Study population

The study included subjects participating in our study on noninvasive hemodynamics, recruiting patients with primary and secondary hypertension and normotensive subjects (clinical trial registration NCT01742702) [26,27]. Written consent was given by all participants. The study complies with the declaration of Helsinki and has been approved by the Tampere University Hospital ethics committee (code R06086M).

We included those participants of the study who had participated in the recording of ambulatory 24-hour BP and laboratory measurements. We excluded subjects with incomplete ambulatory or laboratory recordings and subjects with chronic renal insufficiency stage 4–5 [28]. The present study consisted of 410 participants with a mean age of 50 years and mean BMI of 28 kg/m^2^. Based on ambulatory daytime recordings [1], 69 participants were normotensive and 190 were unmedicated hypertensive patients, and 151 were medicated hypertensive patients (Table 1). The diagnosis and treatment of hypertension were conducted in each subject’s regional primary or secondary health care unit. Altogether 42 patients had secondary hypertension: primary aldosteronism (n = 29), cortisol-induced hypertension (n = 5), renal hypertension (n = 5), and renovascular hypertension (n = 3). Medications used by the participants are presented in Supplementary Table 1, Supplemental digital content 1, http://links.lww.com/BPMJ/A195.

**Table 1:** Basic clinical characteristics, laboratory values, and mean blood pressure during ambulatory daytime recordings in normotensive subjects, unmedicated hypertensive patients, and medicated hypertensive patients

Variable	Normotensive (n = 69)	Unmedicated hypertensive (n = 190)	Medicated hypertensive (n = 151)
Male/female (n)	37/32	118/72	112/39^a^
Age (years)	45 (15)	47 (12)	57 (12)^a^^,^^b^
BMI (kg/m^2^)	25 (4)	28 (5)^a^	29 (5)^a^
Cornell voltage product (ms × mm)	1640 (1000)	1800 (630)	1940 (1000)^a^
Creatinine (µmol/L)	76 (15)	80 (51)	81 (18)
Sodium (mmol/L)	140.9 (2.0)	141.2 (1.8)	140.6 (2.7)
Potassium (mmol/L)	3.7 (0.2)	3.8 (0.3)	3.7 (0.4)
Glucose (mmol/L)	5.4 (0.7)	5.6 (0.6)	6.3 (1.5)^a^^,^^b^
Total cholesterol (mmol/L)	4.8 (1.0)	5.4 (1.0)^a^	5.0 (1.1)^b^
HDL cholesterol (mmol/L)	1.7 (0.5)	1.5 (0.4)^a^	1.4 (0.4)^a^
LDL cholesterol (mmol/L)	2.8 (0.9)	3.5 (0.9)^a^	3.2 (1.0)^b^
Triglycerides (mmol/L)	1.0 (0.5)	1.3 (0.7)^a^	1.4 (0.7)^a^
SBP (mmHg)			
Ambulatory daytime	123 (7)	144 (11)^a^	140 (15)^a^^,^^b^
Laboratory mean(supine+upright)	124 (13)	145 (15)^a^	140 (17)^a^^,^^b^
Diastolic blood pressure (mmHg)			
Daytime diastolic	78 (4)	92 (7)^a^	85 (8)^a^^,^^b^
Laboratory mean(supine+upright)	73 (8)^c^	87 (9)^a^^,^^c^	82 (10)^a^^,^^c^

Laboratory values are calculated from the means of the last 3 min during the 5-minute supine and the last 3 min during the 5-minute head-up tilt recordings; mean (SD).

HDL, high-density lipoprotein; LDL, low-density lipoprotein.

a *P* < 0.05 versus normotensive.

b *P* < 0.05 versus unmedicated hypertensive.

c *P* < 0.05 versus corresponding ambulatory daytime diastolic blood pressure.

### Blood pressure recordings

All participants went through ambulatory, office, and laboratory BP recordings, performed with a median of 7 days apart (25th–75th percentiles 0–18 days). Ambulatory BP recording was performed with Microlife WatchBP O3 (Microlife AG, Widnau, Switzerland) [29] or Mobil-O-Graph (IEM GmbH, Stolberg, Germany) [30]. Daytime BP values were recorded at 20-minute intervals and nighttime at 30-minute intervals. The mean daytime values were applied to the present study. Seated office BP was measured according to the guidelines [1] using a sphygmomanometer (Heine Gamma G7, Herrsching, Germany).

**Fig. 1:**
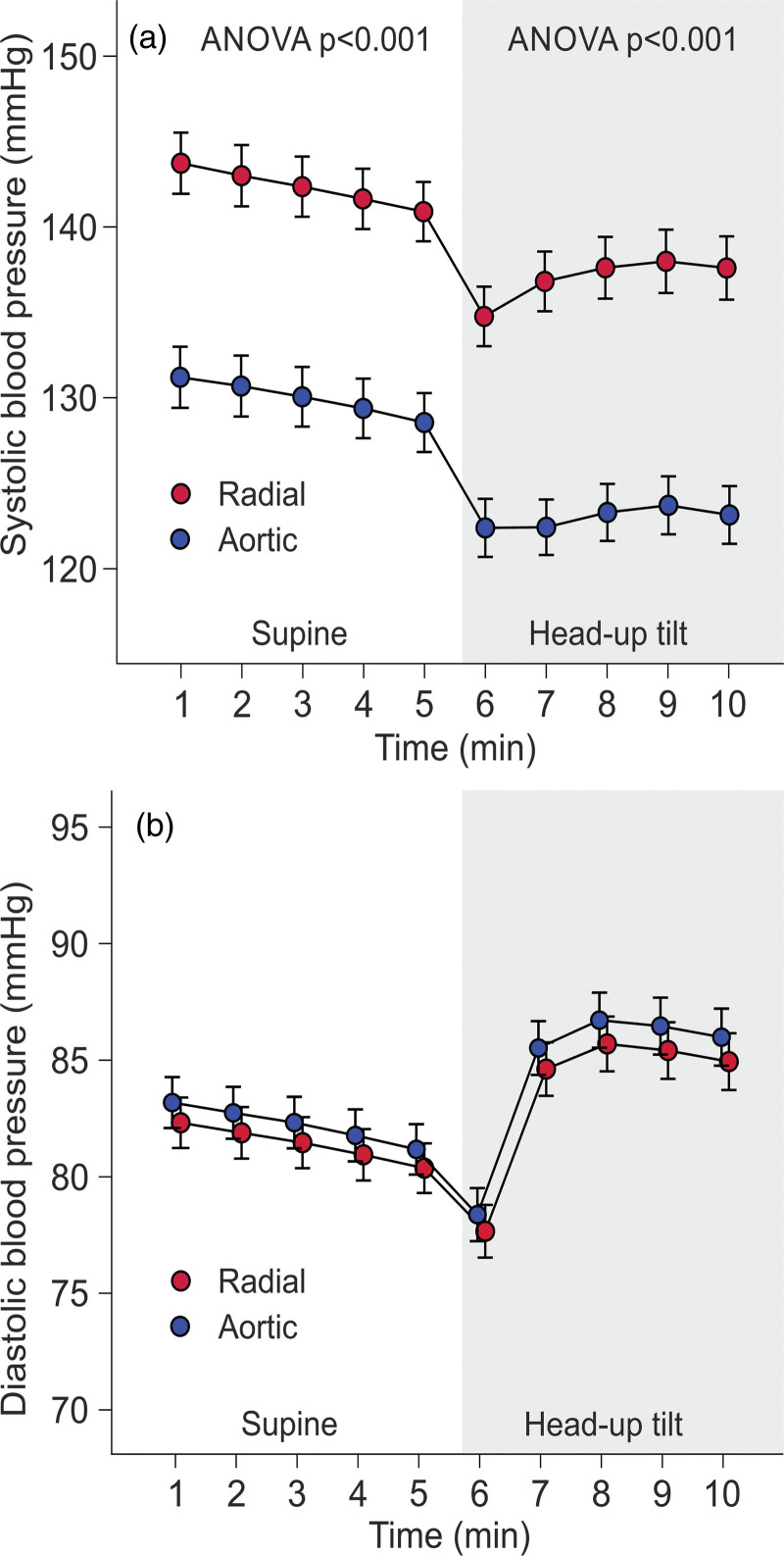
Line graphs show radial and aortic tonometric SBP (a), and DBP (b) in the study population consisting of normotensive subjects (n = 69), unmedicated hypertensive patients (n = 151), and medicated hypertensive patients (n = 190) during 10-minute laboratory recordings; minutes 1–5 in supine position, minutes 6–10 during passive head-up tilt; mean and 95% confidence interval of the mean.

In the laboratory, radial BP from the left wrist was recorded using a tonometric sensor (Colin BP-508T; Colin Medical Instruments Corp., San Antonio, Texas, USA), calibrated approximately every 2.5 min with contralateral brachial BP measurements, coupled with the SphygmoCor PWMx monitoring system (AtCor Medical, Sydney, Australia). Previously, the Colin tonometric device provided accurate data throughout a wide BP range satisfying the Association for the Advancement of Medical Instrumentation standards for mean SBP and DBP measurements but was found to minimally exceed the allowable SD [31]. The subjects were to abstain from smoking, caffeine, and heavy meals for 4 h, and from alcohol for 24 h prior to the recordings. The left arm with the wrist sensor was abducted to 90 degrees in arm support, which held the measurement probe at the heart level in supine and upright positions. Before the measurement, an introductory head-up tilt was performed to familiarize the study subject with the protocol [25]. Aortic BP was derived from the radial BP signal by the SphygmoCor software [13]. Hemodynamic data were recorded for 5 min in the supine position and 5 min during head-up tilt. Mean BP values were calculated for the last 3 min in the supine position, the last 3 min during head-up tilt, and for the average of these 3-minute periods in supine+head-up tilt positions. During the last 3 min in each position, the BP signal was most stable (Fig. 1).

### Laboratory analyses

Blood samples were collected after ~12 h of fasting. Plasma electrolytes, creatinine, C-reactive protein, glucose, and lipid determinations were carried out using Cobas Integra 700/800 (F. Hoffmann-LaRoche Ltd., Basel, Switzerland) or Cobas 6000, module c501 (Roche Diagnostics, Basel, Switzerland). Standard 12-lead electrocardiograms were registered and the Cornell voltage-duration product was calculated [32].

### Statistical analyses

The laboratory results and BP values were analyzed using analysis of variance and calculation of Pearson and Spearman correlations, as appropriate. In some subjects, SBP increased and in some decreased in response to the head-up tilt. Subsequently, the subjects were divided into tertiles according to the change in radial SBP in response to the head-up tilt, and results were analyzed using analysis of variance for repeated measurements. To test whether the laboratory measurements classified subjects similarly to ambulatory BP, the participants were divided into four subgroups: (1) normotensive in ambulatory and laboratory recordings, (2) hypertensive in ambulatory but normotensive in laboratory recordings, (3) normotensive in ambulatory but hypertensive in laboratory recordings, and (4) hypertensive in both recordings. The sensitivity and specificity of the laboratory recordings to define hypertension versus ambulatory and office BP measurements were examined using receiver operating characteristic (ROC) curves. The statistics were performed using SPSS version 26.0 (IBM SPSS, Armonk, New York, USA), and Bonferroni correction was applied in post-hoc analyses. The results in the tables were presented as means and standard deviations of the mean, in the figures as means and 95% confidence intervals of the mean. *P* < 0.05 was considered statistically significant.

## Results

### Study population

In total, 143 (35%) of the subjects were women, and participant ages ranged from 19 to 70 years (Table 1). The medicated hypertensive patients had the highest proportion of males, participant age, and Cornell voltage-duration product (Table 1). The mean BMI and triglyceride concentration were higher, and high-density lipoprotein cholesterol was lower, in unmedicated and medicated hypertensive patients than in normotensive participants. Fasting plasma glucose was highest in medicated hypertensive patients, whereas total and low-density lipoprotein cholesterol levels were highest in unmedicated hypertensive subjects. Plasma creatinine, sodium, and potassium were corresponding in all groups (Table 1). Ambulatory daytime SBP and DBP were highest in unmedicated hypertensive and lowest in normotensive subjects (Table 1). On average, radial and aortic SBP decreased, and DBP increased in response to head-up tilt (Fig. 1).

### Use of mean(supine+upright) blood pressure values in comparisons with ambulatory blood pressure

**Fig. 2:**
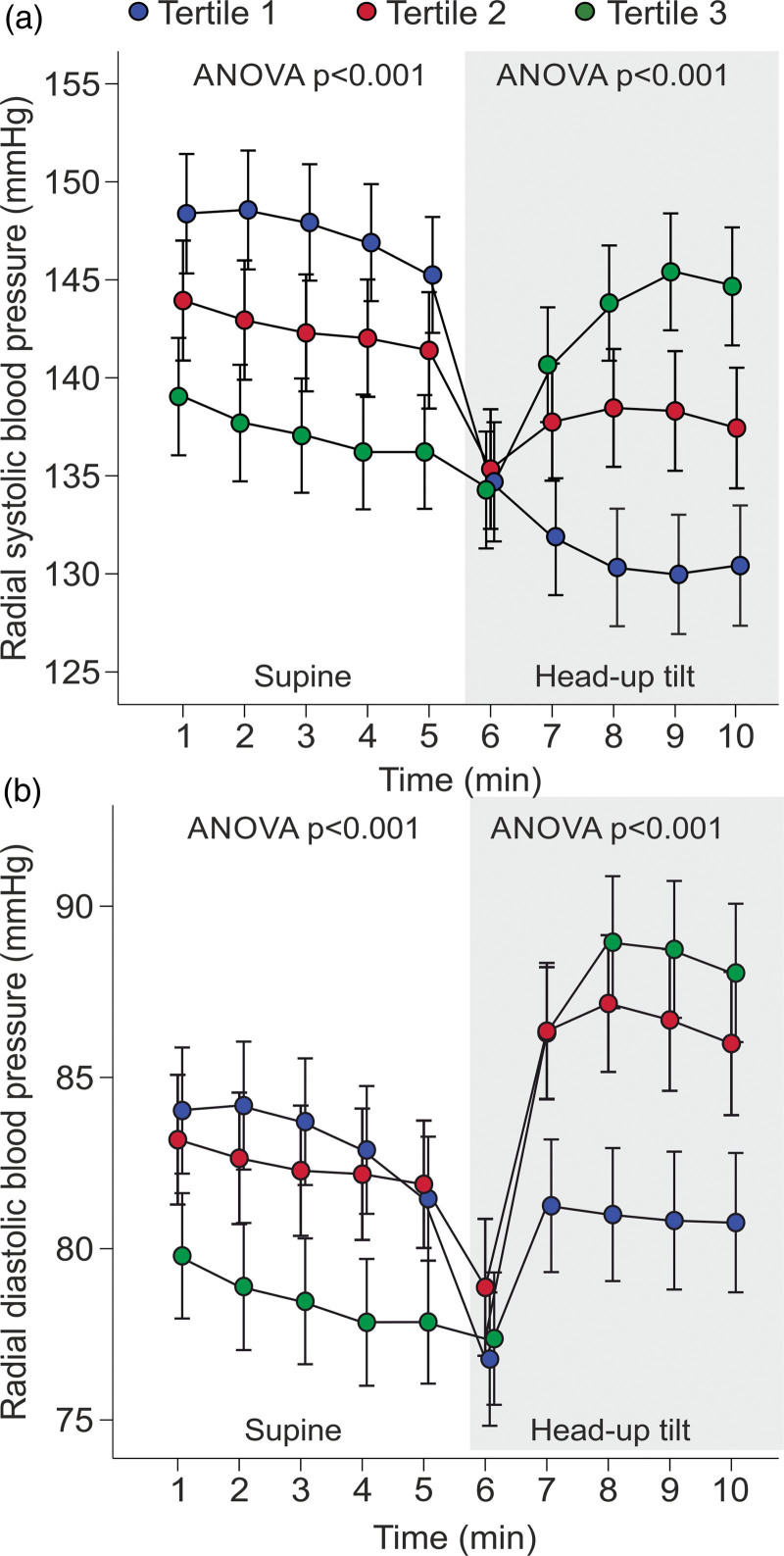
Line graphs show radial tonometric SBP (a) and diastolic blood pressure (b) of study participants divided into tertiles according to the magnitude of the change in radial SBP during head-up tilt; minutes 1–5 in supine position, minutes 6–10 during passive head-up tilt; mean and 95% confidence interval of the mean.

When divided into tertiles according to the change in SBP during head-up tilt, BP either increased or decreased when upright (Fig. 2). Upright SBP decreased in tertiles 1 and 2 and increased in tertile 3; DBP decreased in tertile 1 and increased in tertiles 2 and 3 (*P* < 0.05 for all changes) (Fig. 2). The mean(supine+upright) BP values were applied for the evaluation of the cutoff for hypertension in the laboratory.

Individuals with different BP levels present variations in the white-coat effect [33]. Therefore, we compared ambulatory and laboratory BPs in the normotensive and hypertensive groups. The mean(supine+upright) SBP did not differ from ambulatory daytime SBP in normotensive subjects or unmedicated and medicated hypertensive patients, whereas the mean(supine+upright) DBP was 3–5 mmHg lower than ambulatory DBP in all groups (Table 1).

In the participants, office SBP and DBP were higher, and office heart rate lower than corresponding values during ambulatory BP recordings (Table 2). In the laboratory, supine radial SBP was higher than ambulatory daytime SBP (*P* < 0.05), whereas upright and mean(supine+upright) SBP was corresponding to ambulatory SBP (Table 2). In all laboratory recordings, aortic SBP and radial DBP were lower than ambulatory brachial SBP and DBP values, respectively (*P* < 0.05). Supine and mean(supine+upright) aortic DBP were lower than ambulatory DBP, whereas upright aortic DBP did not differ from ambulatory DBP (Table 2). Heart rate in the office, and in supine and mean(supine+upright) analyses were lower, while during the head-up tilt, heart rate was higher than in ambulatory recordings (*P* < 0.05) (Table 2).

**Table 2:** Mean values of ambulatory daytime blood pressure, office blood pressure, radial and aortic blood pressure, and heart rate

	Ambulatory	Office	Laboratory measurement			
				Supine	Head-up tilt	Mean(supine+upright)
SBP (mmHg)	139 (14)	145 (18)^a^	Radial	142 (18)^a^	138 (18)	140 (17)
			Aortic	129 (18)^a^	123 (17)^a^	126 (16)^a^
DBP mmHg)	87 (9)	91 (12)^a^	Radial	81 (11)^a^	85 (12)^a^	83 (11)^a^
			Aortic	82 (11)^a^	86 (17)	84 (11)^a^
Heart rate (1/min)	71 (10)	67 (10)^a^		63 (9)^a^	75 (11)^a^	69 (10)^a^

Laboratory values are calculated from the means of the last 3 min during the 5-min supine and the last 3 min during the 5-min head-up tilt recordings; mean (SD).

BP, blood pressure.

a *P* < 0.05 versus respective ambulatory BP.

### Evaluation of cutoff values for hypertension in the laboratory versus ambulatory recordings

**Fig. 3:**
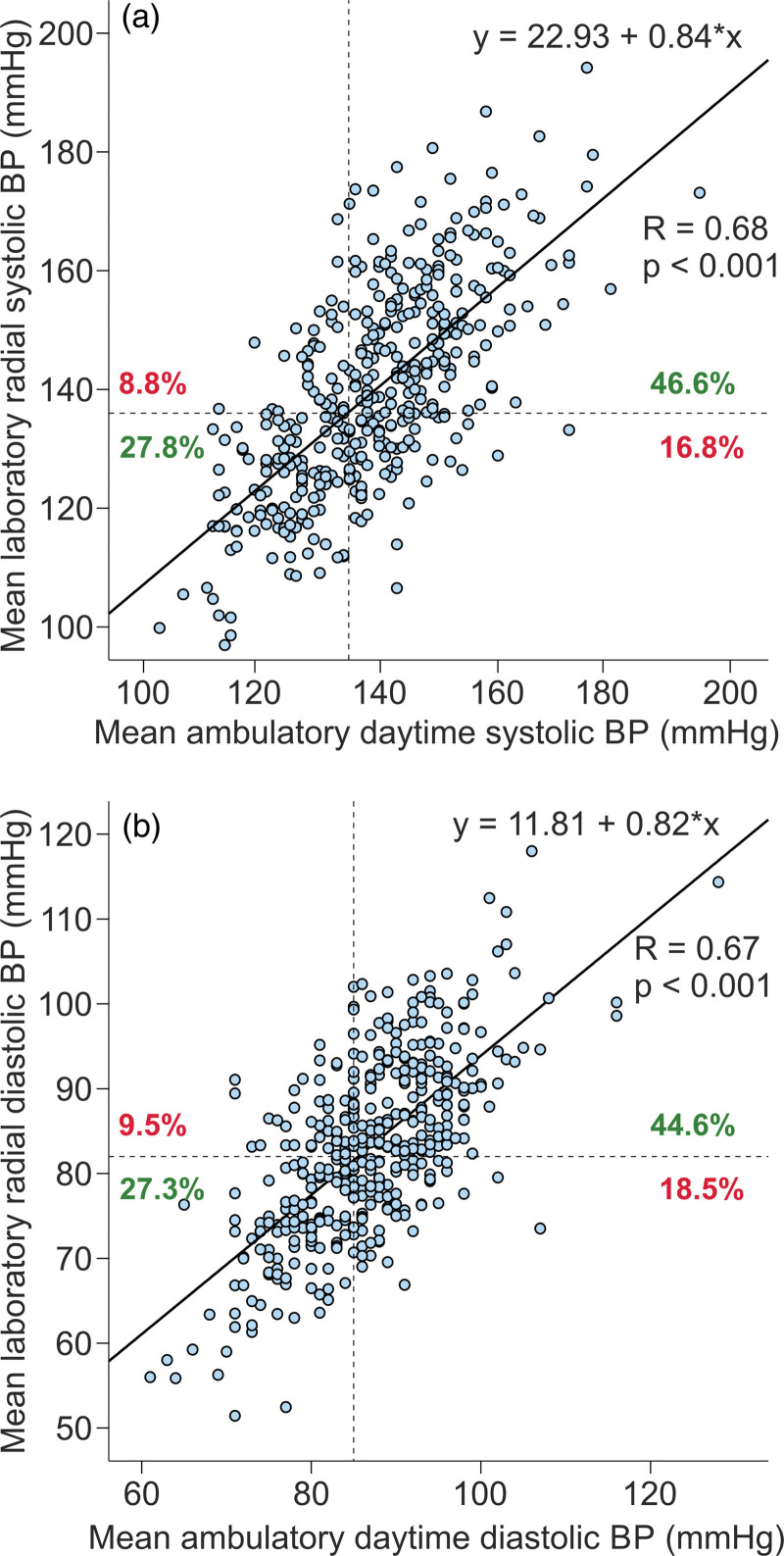
Correlograms of mean ambulatory daytime blood pressure (BP) versus mean(supine+upright) radial tonometric BP; SBP (a), DBP (b), dotted black lines represent cutoff points for hypertension, green numbers denote the proportion of subjects classified similarly, and red numbers denote the proportion of subjects classified dissimilarly to hypertensive and normotensive subjects.

Scatter plots of ambulatory daytime BP versus mean (supine+upright) radial tonometric BP are shown in Fig. 3. The line graphs in the scatter plots suggested that i) systolic laboratory BP 136 mmHg corresponded to ambulatory 135 mmHg, and ii) diastolic laboratory BP 82 mmHg corresponded to ambulatory 85 mmHg (Fig. 3). Respectively, the corresponding cutoff to define hypertension in the aortic recordings was 123/83 mmHg.

With the above cutoffs, 311 (76%) of the participants were classified similarly in ambulatory and laboratory recordings: 76 (19%) subjects were normotensive and 235 (57%) were hypertensive (Table 3). Sixty-eight (17%) patients were hypertensive only during ambulatory monitoring but had normal laboratory BP. Altogether 24 of these 68 subjects had normal BP in the office indicating masked hypertension. Moreover, 31 (8%) subjects were hypertensive only during tonometric radial artery recordings, but their ambulatory values were normal (Table 3). Altogether 19 of these 31 subjects were hypertensive also in the office indicating white-coat hypertension.

**Table 3:** Classification of subjects to hypertensive and normotensive groups when applying the radial cut-point 136/82 mmHg in laboratory measurements: 311 (75.8%) of the subjects were classified similarly in ambulatory and laboratory recordings

	Normotensive (n = 76)	Hypertensive in ABP daytime only (n = 68)	Hypertensive in laboratory only (n = 31)	Hypertensive in both ABP daytime and laboratory (n = 235)
Mean systolic ABP daytime (mmHg)	122 (7)	138 (9)^a^	127 (6)^a^^,^^b^	147 (11)^a^^,^^b^^,^^c^
Mean diastolic ABP daytime (mmHg)	77 (5)	88 (6)^a^	80 (3)^a^^,^^b^	91 (8)^a^^,^^b^^,^^c^
Laboratory radial SBP mean(supine+upright) (mmHg)	120 (10)	127 (7)^a^	139 (9)^a^^,^^b^	150 (13)^a^^,^^b^^,^^c^
Laboratory radial DBP mean(supine+upright) (mmHg)	71 (7)	76 (4)^a^	83 (5)^a^^,^^b^	89 (9)^a^^,^^b^^,^^c^
Laboratory aortic SBP mean(supine+upright) (mmHg)	107 (9)	115 (7)	125 (10)^a^^,^^b^	136 (13)^a^^,^^b^^,^^c^
Laboratory aortic DBP mean(supine+upright) (mmHg)	72 (7)	77 (4)	84 (5)^a^	90 (9)^a^^,^^b^

Laboratory values are calculated from the means of the last 3 min during the 5-min supine and the last 3 min during the 5-min head-up tilt recordings; mean (SD).

ABP, ambulatory blood pressure.

a *P* < 0.05 versus normotensive.

b *P* < 0.05 versus hypertensive in ABP.

c *P* < 0.05 versus hypertensive in laboratory. When applying the aortic BP cutoff point 123/83 mmHg, 313 (76.3%) of the subjects were classified similarly in ambulatory and laboratory recordings.

**Fig. 4:**
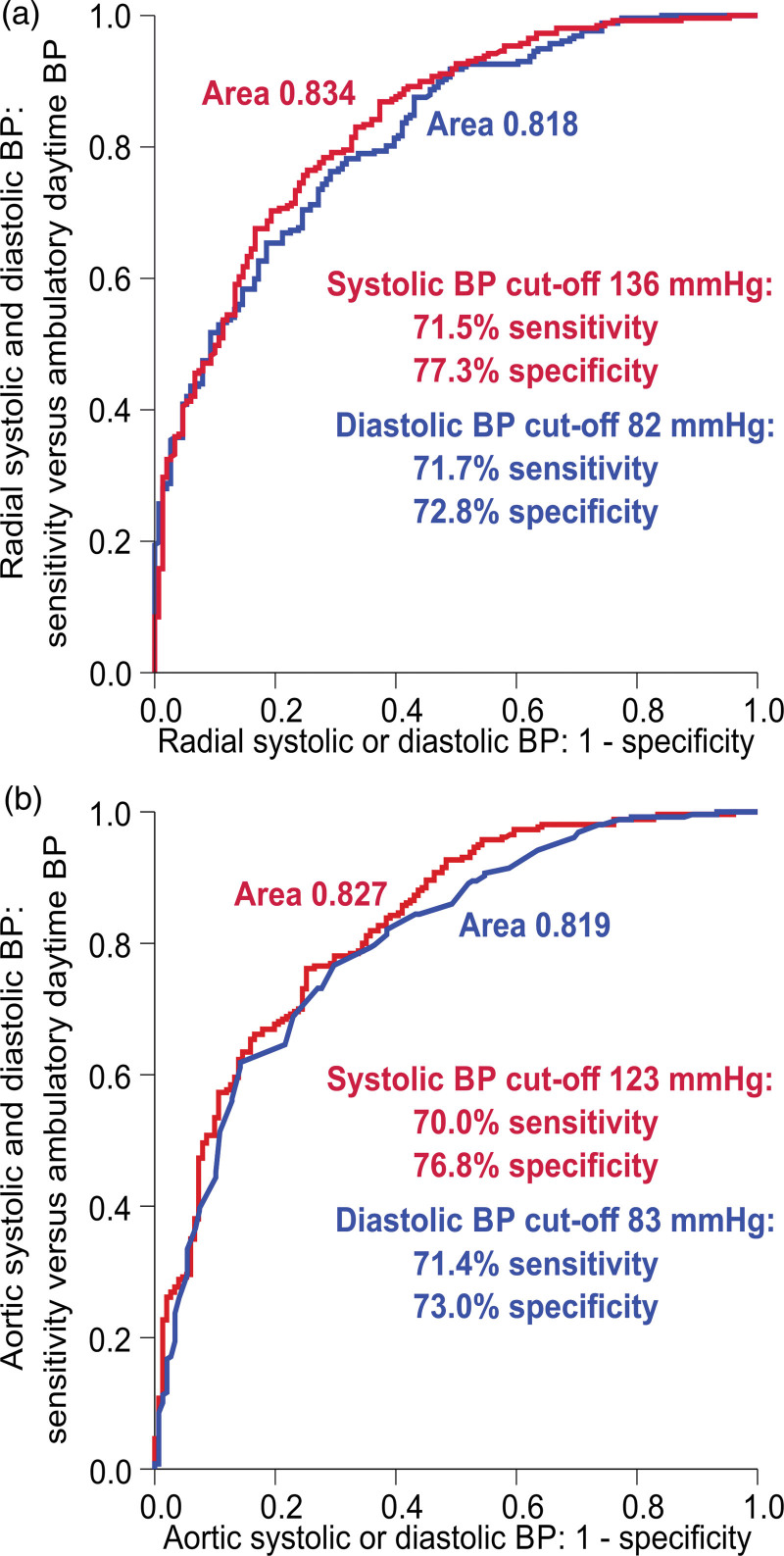
Receiver operating characteristic curves depicting the prediction of brachial ambulatory blood pressure (BP) ≥135/85 mmHg by the mean(supine+upright) radial tonometric SBP (red line) and DBP (red line) (a), and by the mean(supine+upright) aortic SBP (red line) and DBP (red line) (b).

ROC curve analyses of the radial cutoff 136/82 mmHg and the aortic cutoff 123/83 mmHg versus the ambulatory cutoff 135/85 mmHg are presented in Fig. 4. The laboratory radial systolic cutoff 136 mmHg had a sensitivity of 71.5% and specificity of 77.3%, and the laboratory radial diastolic cutoff 82 mmHg had a sensitivity of 71.7% and specificity of 72.8% to define hypertension (Fig. 4a). The laboratory aortic systolic cutoff 123 mmHg had a sensitivity of 70.0% and specificity of 76.8%, and the laboratory aortic diastolic cutoff 83 mmHg had a sensitivity of 71.4% and specificity of 73.0% to define hypertension (Fig. 4b). Additionally, when compared with the office cutoff 140/90 mmHg, the laboratory radial SBP cutoff 136 mmHg had a sensitivity of 71.4% and specificity of 75.8%, and the laboratory radial DBP cutoff 82 mmHg had a sensitivity of 71.4% and specificity of 68.4% to define hypertension.

The correlations between laboratory and ambulatory BPs were also analyzed separately in subjects who had never received BP-lowering medications and in patients treated with antihypertensive agents. The correlations of these approaches for SBP were 0.68 in the unmedicated group and 0.69 in medicated group, and for DBP 0.70 in the unmedicated group and 0.61 in the medicated group. These results indicate that antihypertensive medications were not a source of confounding in the present study.

## Discussion

A high proportion of the active human life is spent in the upright position, while the hemodynamic responses to upright posture are influenced e.g. by antihypertensive medication [34], liquorice intake [9], cardiovascular disease [24], age [25], and sex [18]. Here we compared radial tonometric BP status during head-up tilt versus ambulatory daytime BP, and evaluated the effect of body posture on BP. As a significant proportion of research regarding the hemodynamics of hypertension has been performed under laboratory conditions [10,11,35,36], we evaluated a potential cutoff point for hypertension during tonometric laboratory recordings. We stress that the present results should not be considered as an attempt to find a novel approach for the diagnosis of hypertension but as an effort to compare the tonometric method with ambulatory daytime brachial BP for research purposes. The study population was representative, as it consisted of men and women, normotensive subjects, and unmedicated and medicated hypertensive patients. The hypertensive groups had higher BMI and worse lipids than the normotensive subjects [1], while the medicated hypertensive patients were the oldest, had the highest plasma glucose, and the highest proportion of males.

According to the correlograms of ambulatory daytime BP versus mean(supine+upright) radial tonometric BP, the laboratory BP 136/82 mmHg corresponded to ambulatory daytime BP 135/85 mmHg. Previously, no reference values for hypertension have been defined for noninvasive laboratory recordings [12,18,35]. BP is characterized by high variability depending on measuring conditions, age, stressor factors, and body position [1,3,5,11,12]. As the mean resting ambulatory heart rate in >92 000 individuals was 65/min [2], there are ~100 000 SBP and DBP values within 24 h, and the definition of the cutoff for hypertension is not straightforward.

Radial SBP and pulse pressure are higher than the aortic values due to pulse pressure amplification [37]. Recently, four different BP phenotypes of SBP amplification were discovered: amplification of both aortic to brachial and brachial to radial SBP, only aortic to brachial amplification, only brachial to radial amplification, and neither aortic to brachial nor brachial to radial amplification [37]. The latter two phenotypes had higher aortic BP, which could not be differentiated using standard cuff measurements [37]. The central hemodynamic variables, including aortic pulse pressure and pulse wave velocity, predict cardiovascular risk better than brachial measurements [15,16]. Several variables reflecting central circulation can be derived from the peripheral tonometric signal [9–12,18], but the tonometric BP recording is sensitive to measurement errors, for example, due to upper arm movements during the recordings [38].

Weiss *et al*. considered the accuracy of tonometric BP values to be moderate in 22 surgical patients [38], while Steiner *et al*. found that mean BP values differed in ~34% of the readings by >10 mmHg from intra-arterial readings among 15 neuro-intensive care patients [39]. However, Kemmotsu *et al*. reported that tonometry provided accurate and reliable monitoring of BP in 28 patients undergoing orthopedic surgery [40], while Nelesen and Dimsdale found that radial tonometric BP monitoring had low artifact rating and high accuracy during stressor tests among 20 subjects when compared with noninvasive recordings using Dinamap and Finapres [41]. In the present study, we calculated average BPs during 1-minute-long and 3-minute-long recording periods from a much larger group of 410 participants to increase the reliability of the measurements. Of note, the pulse waveforms were captured from the radial artery, but the signal was calibrated by oscillometric cuff-based measurements from the contralateral brachial artery. Therefore, the actual comparison of BP levels was between radial tonometric signal calibrated from brachial BP versus ambulatory daytime brachial BP.

In the present study, office BP was higher than ambulatory BP in each study group. Higher ambulatory daytime than office heart rate can be explained by out-of-office physical activity. Our laboratory recordings confirmed that body position has a significant effect on the level of BP [10,12,18,42]. On average, SBP decreased and diastolic increased during head-up tilt [18,42]. However, as shown in Fig. 2, the response to head-up tilt was variable, and SBP and DBP either increased or decreased. Therefore, we applied the mean(supine+upright) BP in the analyses. Using this approach, the tonometric SBP did not significantly differ, while the tonometric DBP was lower in the laboratory recordings versus ambulatory recordings. Moreover, an active change of body position from supine or seated to standing requires muscle work, which influences hemodynamics. In a recent report, systolic aortic BP did not change, while an increase was observed in aortic DBP when examined in supine, seated, and standing postures in 426 normotensive 21-year-old subjects [43]. Passive head-up tilt under laboratory circumstances minimizes the influences of external stressor factors on hemodynamics.

We recently examined hemodynamic differences underlying the various BP responses to head-up tilt in 613 participants without the cardiovascular disease [44]. One-third of the participants presented with higher upright than supine SBP which was explained by differences in the regulation of systemic vascular resistance and cardiac output [44]. When we compared hemodynamic responses to head-up tilt in hypertensive versus normotensive subjects, the principal differences were higher systemic vascular resistance and increased large artery stiffness in hypertensive patients [12]. However, there were several subtle differences between the groups in response to the head-up tilt: aortic SBP, pulse pressure, and left cardiac work index decreased less; heart rate increased less; and aortic DBP increased more in hypertensive patients. Thus, functional alterations beyond increased vascular resistance and arterial stiffness are present in essential hypertension [12].

An important and reliable method in the diagnosis of hypertension is ambulatory BP monitoring [1]. Also, repeated home measurements are considered more reliable than office BP measurements [1]. The common phenotypes of high BP, white-coat, and masked hypertension [1], exemplify that a single approach can give false information about BP. As shown in Table 3, about 76% of the present study subjects were classified similarly as normotensive or hypertensive in the tonometric versus ambulatory recordings. However, 68 participants were hypertensive only during ambulatory daytime monitoring and 31 only during laboratory recordings. Elevated BP exclusively in the ambulatory monitoring may be attributed to masked hypertension or higher physical activity during the recording, and the office measurements suggest that 24 of the 68 participants (35%) had masked hypertension. Elevated BP exclusively in the laboratory may be explained by white-coat hypertension and based on office measurements 19 of the 31 (61%) had white-coat hypertension.

Of note, subjects classified as hypertensive in ambulatory recordings only were clearly normotensive in the laboratory and their aortic BP did not differ from the group that was normotensive in both ambulatory and tonometric recordings. Furthermore, subjects classified as hypertensive in the laboratory only had higher aortic BP than subjects who were normotensive in both ambulatory and tonometric recordings, and their diastolic aortic BP did not differ from the group that was hypertensive in both tonometric and ambulatory recordings (Table 3).

The ROC curves showed that the laboratory and ambulatory BP values were not entirely corresponding (Fig. 4). Moreover, the 136/82 mmHg laboratory BP cutoff did not quite correspond to the office BP cutoff of 140/90 mmHg, either. The deviations between various approaches to measuring BP can be attributed to the distinct BP reactions of individuals to variations in the measuring conditions and methods. Altogether, the sensitivity and specificity of all present approaches may be suboptimal due to the high number of unknown BP values within a single day. The limitations concerning the accuracy of indirect BP measurements [3,4] also apply to the present cross-sectional study as the tonometric recordings were calibrated using brachial BP measurements. The results of the present study may also be influenced by interarm differences in BP. Clark *et al*. examined 230 hypertensive patients (mean age of 68 years) and found that an interarm difference in office SBP of ≥10 mmHg was associated with >2-fold hazard ratio for cardiovascular events and >3-fold hazard ratio for mortality during 10 years of follow-up [45]. On the other hand, in this elderly population mean SBP was 1.5 mmHg higher in the right arm, while the mean DBP was 1.7 mmHg higher in the left arm [45]. Thus, on average the interarm differences in BP were not major, and BP calibrated from the right arm can be assumed to reflect BP in the left arm rather well.

### Conclusions

As most of the hemodynamic research has been performed in the laboratory, we compared tonometric laboratory BP recordings with ambulatory daytime BP. High individual variation in the BP in response to head-up tilt suggests that upright measurements along with supine or seated measurements should be considered in the evaluation of the BP level. Seated BP measurements are the prevailing standard for BP measurements both in the office and at home [1]. The present results implied that mean(supine+upright) laboratory BP 136/82 mmHg corresponded to mean daytime ambulatory BP 135/85 mmHg, and 76% of the participants who were either hypertensive or normotensive were classified similarly using both approaches. However, BP status in the remaining 24% of subjects, among whom significant proportions were white-coat or masked hypertensive, more depended on the measuring conditions, which were probably influenced by higher physical activity during out-of-office recordings.

## Acknowledgements

The authors thank research nurses Emmi Hirvelä, Paula Erkkilä, and Reeta Kulmala, for invaluable technical assistance. The authors wish to acknowledge CSC – IT Center for Science, Finland, for computational resources.

This work was supported by Competitive State Research Financing of the Expert Responsibility Area of Tampere University Hospital (grants 9AA062 and 9AB057); Finnish Foundation for Cardiovascular Research; Päivikki and Sakari Sohlberg Foundation, Sigrid Jusélius Foundation, Pirkanmaa Regional Fund of the Finnish Cultural Foundation, Pirkanmaa Regional Society of the Finnish Kidney and Liver Association, and The Finnish Medical Foundation.

Data availability statement: Analyses and datasets of the current study are not available publicly as the clinical database contains indirect identifiers and the informed consent does not allow publication of individual patient data. The datasets are available from the corresponding author upon reasonable request.

### Conflicts of interest

There are no conflicts of interest.

## Supplementary Material



## References

[1] Williams B Mancia G Spiering W Agabiti Rosei E Azizi M Burnier M.; ESC Scientific Document Group. 2018 ESC/ESH guidelines for the management of arterial hypertension. Eur Heart J 2018; 39:3021–3104. 30165516 10.1093/eurheartj/ehy339

[2] Quer G Gouda P Galarnyk M Topol EJ Steinhubl SR. Inter- and intraindividual variability in daily resting heart rate and its associations with age, sex, sleep, BMI, and time of year: Retrospective, longitudinal cohort study of 92,457 adults. PLoS One 2020; 15:e0227709. 32023264 10.1371/journal.pone.0227709 PMC7001906

[3] Picone DS Schultz MG Otahal P Black JA Bos WJ Chen C-H.; Invasive Blood Pressure Consortium. Influence of age on upper arm cuff blood pressure measurement. Hypertension 2020; 75:844–850. 31983305 10.1161/HYPERTENSIONAHA.119.13973 PMC7035100

[4] Picone DS Schultz MG Otahal P Aakhus S Al-Jumaily AM Black JA. Accuracy of cuff-measured blood pressure. J Am Coll Cardiol 2017; 70:572–586. 28750701 10.1016/j.jacc.2017.05.064

[5] Pierdomenico SD Pierdomenico AM Coccina F Clement DL De Buyzere ML De Bacquer DA. Prognostic value of masked uncontrolled hypertension: systematic review and meta-analysis. Hypertension 2018; 72:862–869. 30354717 10.1161/HYPERTENSIONAHA.118.11499 PMC6205750

[6] Huang Y Huang W Mai W Cai X An D Liu Z. White-coat hypertension is a risk factor for cardiovascular diseases and total mortality. J Hypertens 2017; 35:677–688. 28253216 10.1097/HJH.0000000000001226 PMC5338886

[7] Forouzanfar MH Liu P Roth GA Ng M Biryukov S Marczak L. Global burden of hypertension and systolic blood pressure of at least 110 to 115 mmHg, 1990-2015. JAMA 2017; 317:165–182. 28097354 10.1001/jama.2016.19043

[8] Whelton SP McEvoy JW Shaw L Psaty BM Lima JAC Budoff M. Association of normal systolic blood pressure level with cardiovascular disease in the absence of risk factors. JAMA Cardiol 2020; 5:1011–1018. 32936272 10.1001/jamacardio.2020.1731 PMC7287937

[9] Hautaniemi EJ Tahvanainen AM Koskela JK Tikkakoski AJ Kähönen M Uitto M. Voluntary liquorice ingestion increases blood pressure via increased volume load, elevated peripheral arterial resistance, and decreased aortic compliance. Sci Rep 2017; 7:10947. 28887501 10.1038/s41598-017-11468-7 PMC5591274

[10] Suojanen L Haring A Tikkakoski A Huhtala H Kähönen M Eräranta A. Adverse influence of bisoprolol on central blood pressure in the upright position: a double-blind placebo-controlled cross-over study. J Hum Hypertens 2020; 34:301–310. 30886326 10.1038/s41371-019-0188-9 PMC7165126

[11] Kangas P Tikkakoski A Kettunen J Eräranta A Huhtala H Kähönen M. Changes in hemodynamics associated with metabolic syndrome are more pronounced in women than in men. Sci Rep 2019; 9:18377. 31804574 10.1038/s41598-019-54926-0 PMC6895092

[12] Tikkakoski AJ Tahvanainen AM Leskinen MH Koskela JK Haring A Viitala J. Hemodynamic alterations in hypertensive patients at rest and during passive head-up tilt. J Hypertens 2013; 31:906–915. 23412427 10.1097/HJH.0b013e32835ed605

[13] Chen CH Nevo E Fetics B Pak PH Yin FC Maughan WL. Estimation of central aortic pressure waveform by mathematical transformation of radial tonometry pressure. Validation of generalized transfer function. Circulation 1997; 95:1827–1836. 9107170 10.1161/01.cir.95.7.1827

[14] Hamid H Kurra V Choudhary MK Bouquin H Niemelä O Kähönen MAP. Plasma uric acid is related to large arterial stiffness but not to other hemodynamic variables: a study in 606 normotensive and never-medicated hypertensive subjects. BMC Cardiovasc Disord 2021; 21:257. 34039285 10.1186/s12872-021-02072-9 PMC8152327

[15] Niiranen TJ Kalesan B Hamburg NM Benjamin EJ Mitchell GF Vasan RS. Relative contributions of arterial stiffness and hypertension to cardiovascular disease: the Framingham heart study. J Am Heart Assoc 2016; 5:e004271. 27912210 10.1161/JAHA.116.004271 PMC5210358

[16] Vlachopoulos C Aznaouridis K O’Rourke MF Safar ME Baou K Stefanadis C. Prediction of cardiovascular events and all-cause mortality with central haemodynamics: a systematic review and meta-analysis. Eur Heart J 2010; 31:1865–1871. 20197424 10.1093/eurheartj/ehq024

[17] Tahvanainen AM Tikkakoski AJ Koskela JK Nordhausen K Viitala JM Leskinen MH. The type of the functional cardiovascular response to upright posture is associated with arterial stiffness: a cross-sectional study in 470 volunteers. BMC Cardiovasc Disord 2016; 16:101. 27216309 10.1186/s12872-016-0281-8 PMC4877753

[18] Kangas P Tahvanainen A Tikkakoski A Koskela J Uitto M Viik J. Increased cardiac workload in the upright posture in men: noninvasive hemodynamics in men versus women. J Am Heart Assoc 2016; 5:e002883. 27329447 10.1161/JAHA.115.002883 PMC4937251

[19] Finucane C O’Connell MDL Fan CW. Age-related normative changes in phasic orthostatic blood pressure in a large population study. Circulation 2014; 130:1780–1789. 25278101 10.1161/CIRCULATIONAHA.114.009831

[20] van Wijnen VK Finucane C Harms MPM Nolan H Freeman RL Westerhof BE. Noninvasive beat-to-beat finger arterial pressure monitoring during orthostasis: a comprehensive review of normal and abnormal responses at different ages. J Intern Med 2017; 282:468–483. 28564488 10.1111/joim.12636

[21] Nardo CJ Chambless LE Light KC Rosamond WD Sharrett AR Tell GS. Descriptive epidemiology of blood pressure response to change in body position: the ARIC study. Hypertension 1999; 33:1123–1129. 10334798 10.1161/01.hyp.33.5.1123

[22] Magkas N Tsioufis C Thomopoulos C Dilaveris P Georgiopoulos G Doumas M. Orthostatic hypertension: from pathophysiology to clinical applications and therapeutic considerations. J Clin Hypertens 2019; 21:426–433. 10.1111/jch.13491 PMC8030346 30724451

[23] Jordan J Ricci F Hoffmann F Hamrefors V Fedorowski A. Orthostatic hypertension: critical appraisal of an overlooked condition. Hypertension 2020; 75:1151–1158. 32223382 10.1161/HYPERTENSIONAHA.120.14340

[24] Xin W Mi S Lin Z Wang H Wei W. Orthostatic hypotension and the risk of incidental cardiovascular diseases: a meta-analysis of prospective cohort studies. Prev Med 2016; 85:90–97. 26825758 10.1016/j.ypmed.2016.01.007

[25] Tahvanainen A Leskinen M Koskela J Ilveskoski E Nordhausen K Oja H. Ageing and cardiovascular responses to head-up tilt in healthy subjects. Atherosclerosis 2009; 207:445–451. 19580971 10.1016/j.atherosclerosis.2009.06.001

[26] Oinonen L Koskela J Eräranta A Tikkakoski A Kähönen M Niemelä O. Plasma total calcium concentration is associated with blood pressure and systemic vascular resistance in normotensive and never-treated hypertensive subjects. Blood Press 2020; 29:137–148. 31790289 10.1080/08037051.2019.1696180

[27] Choudhary MK Värri E Matikainen N Koskela J Tikkakoski AJ Kähönen M. Primary aldosteronism: higher volume load, cardiac output and arterial stiffness than in essential hypertension. J Intern Med 2021; 289:29–41. 32463949 10.1111/joim.13115

[28] KDIGO. 2012 Clinical practice guideline for the evaluation and management of chronic kidney disease. Kidney Int Supp 2013; 3:1–150. 10.1038/ki.2013.243 23989362

[29] Ragazzo F Saladini F Palatini P. Validation of the Microlife WatchBP O3 device for clinic, home, and ambulatory blood pressure measurement, according to the International Protocol. Blood Press Monit 2010; 15:59–62. 20075717 10.1097/MBP.0b013e32833531ca

[30] Wei W Tölle M Zidek W van der Giet M. Validation of the mobil-O-graph: 24 h-blood pressure measurement device. Blood Press Monit 2010; 15:225–228. 20216407 10.1097/MBP.0b013e328338892f

[31] Zorn EA Wilson MB Angel JJ Zanella J Alpert BS. Validation of an automated arterial tonometry monitor using Association for the Advancement of Medical Instrumentation standards. Blood Press Monit 1997; 2:185–188. 10234114

[32] Dahlöf B Devereux RB Julius S Kjeldsen SE Beevers G de Faire U. Characteristics of 9194 patients with left ventricular hypertrophy: the LIFE study. Losartan Intervention For Endpoint Reduction in Hypertension. Hypertension 1998; 32:989–997. 9856962 10.1161/01.hyp.32.6.989

[33] Niiranen TJ Jula AM Kantola IM Reunanen A. Prevalence and determinants of isolated clinic hypertension in the Finnish population: the Finn-HOME study. J Hypertens 2006; 24:463–470. 16467649 10.1097/01.hjh.0000209982.21112.bc

[34] Suojanen L Haring A Tikkakoski A Huhtala H Kähönen M Eräranta A. Adverse influence of bisoprolol on central blood pressure in the upright position: a double-blind placebo-controlled cross-over study. J Hum Hypertens 2019; 1:10. 10.1038/s41371-019-0188-9 PMC7165126 30886326

[35] Teodorovich N Swissa M. Tilt table test today - state of the art. WJC 2016; 8:277. 27022459 10.4330/wjc.v8.i3.277 PMC4807316

[36] Gizdulich P Prentza A Wesseling KH. Models of brachial to finger pulse wave distortion and pressure decrement. Cardiovasc Res 1997; 33:698–705. 9093542 10.1016/s0008-6363(97)00003-5

[37] Picone DS Schultz MG Peng X Black JA Dwyer N Roberts-Thomson P. Discovery of new blood pressure phenotypes and relation to accuracy of cuff devices used in daily clinical practice novelty and significance. Hypertension 2018; 71:1239–1247. 29632105 10.1161/HYPERTENSIONAHA.117.10696

[38] Weiss BM Spahn DR Rahmig H Rohling R Pasch T. Radial artery tonometry: moderately accurate but unpredictable technique of continuous non-invasive arterial pressure measurement. Br J Anaesth 1996; 76:405–411. 8785142 10.1093/bja/76.3.405

[39] Steiner LA Johnston AJ Salvador R Czosnyka M Menon DK. Validation of a tonometric noninvasive arterial blood pressure monitor in the intensive care setting. Anaesthesia 2003; 58:448–454. 12694001 10.1046/j.1365-2044.2003.03122.x

[40] Kemmotsu O Ueda M Otsuka H Yamamura T Okamura A Ishikawa T. Blood pressure measurement by arterial tonometry in controlled hypotension. Anesth Analg 1991; 73:54–58. 1858991 10.1213/00000539-199107000-00011

[41] Nelesen RA Dimsdale JE. Use of radial arterial tonometric continuous blood pressure measurement in cardiovascular reactivity studies. Blood Press Monit 2002; 7:259–263. 12409884 10.1097/00126097-200210000-00002

[42] Cicolini G Pizzi C Palma E Bucci M Schioppa F Mezzetti A. Differences in blood pressure by body position (supine, fowler’s, and sitting) in hypertensive subjects. Am J Hypertens 2011; 24:1073–1079. 21677699 10.1038/ajh.2011.106

[43] Choudhary MK Penninkangas R Eräranta A Niemelä O Mangani C Maleta K. Posture‐related differences in cardiovascular function between young men and women: study of noninvasive hemodynamics in rural Malawi. JAHA 2022; 11:e022979. 35195013 10.1161/JAHA.121.022979 PMC9075090

[44] Suojanen LJ Koskela JK Wilenius M Choudhary MK Hautaniemi EJ Viik J. Individual changes of central blood pressure in response to upright posture: different hemodynamic phenotypes. J Hypertens 2021; 39:2403–2412. 34269331 10.1097/HJH.0000000000002965

[45] Clark CE Taylor RS Shore AC Campbell JL. The difference in blood pressure readings between arms and survival: primary care cohort study. BMJ 2012; 344:e1327. 22433975 10.1136/bmj.e1327 PMC3309155

