# Supplementary material for: Ambulatory daytime blood pressure versus tonometric blood pressure measurements in the laboratory: effect of posture
Source: Blood Press Monit. 2023 Jun 8;28(4):199–207. doi: 10.1097/MBP.0000000000000651 (PMC10309093; doi:10.1097/MBP.0000000000000651)
Supplement: Supplementary file 1 [file bpmj-28-199-s001.pdf]

**Supplementary Table S1**. Number of subjects using antihypertensive agents and other medications.

| Antihypertensive medications | Number of subjects |
|---|---|
| Calcium channel blockers | 83 |
| Beta blockers | 75 |
| Angiotensin II receptor blockers | 68 |
| Thiazide diuretics | 60 |
| Angiotensin converting enzyme inhibitors | 45 |
| Potassium sparing diuretics | 21 |
| Prazocin | 17 |
| Moxonidine | 9 |
| Furosemide | 7 |
| Beta + alpha blockers | 3 |
| Minoxidil | 1 |
| **Other medications** | **Number of subjects** |
| Vitamin D | 64 |
| Low acetylsalisylic acid | 49 |
| Antidepressant | 36 |
| Female hormones | 35 |
| Metformin | 23 |
| Proton pump inhibitor | 22 |
| Calcium supplement | 22 |
| Thyroxin* | 22 |
| Potassium supplement | 18 |
| Inhaled corticosteroids | 17 |
| Antihistamine | 13 |
| Tamsulosin of alfuzosin | 13 |
| Antirheumatic drugs | 11 |
| Insulin | 10 |
| Inhaled long-acting beta-agonist | 9 |
| Magnesium supplement | 9 |
| Oral anticoalgulants | 8 |
| Benzodiazepine | 7 |
| Dipeptidyl peptidase-4 inhibitor | 7 |
| Allopurinol | 7 |
| Gabapentin or pregabalin | 6 |
| 5-alpha reductase inhibitor | 6 |
| Intrauterine device | 5 |
| Nonsteroidal anti-inflammatory drug | 5 |
| Glaucoma medication | 4 |

| | |
|---|---|
| Ezetimibe | 3 |
| Glucagon-like peptide-1 agonist | 3 |
| Antiepileptic | 3 |
| Opioid | 3 |
| Sulphonyl urea | 2 |
| Triiodothyronine | 2 |
| Quetiapine | 2 |
| Clopidogrel | 2 |
| Fibrate | 1 |
| Sodium-glucose co-transporter-2 inhibitor | 1 |
| Amiodarone | 1 |
| Oxybutynin | 1 |
| Letrozole | 1 |
| Hydroxycarbamide | 1 |
| Dipyridamole | 1 |
| Aripiprazole | 1 |
| Levomepromazine | 1 |
| Pramipexol | 1 |
| Prednisolone | 1 |

*All subjects were euthyroid
